# Continuous assurance for AI-driven clinical decision support systems

**DOI:** 10.3389/frai.2026.1870819

**Published:** 2026-07-29

**Authors:** Rami A. Al-Horani, Amanuel F. Tadesse

**Affiliations:** 1 Division of Pharmaceutical Sciences, College of Pharmacy, Xavier University of Louisiana, New Orleans, LA, United States; 2 Department of Accounting, LSU New Orleans, Henry Bernstein College of Business Administration, New Orleans, LA, United States

**Keywords:** collaborative decision making, continuous assurance, healthcare artificial intelligence, socio-technical resilience, STRAICS framework

## Abstract

Healthcare systems are rapidly embedding adaptive and generative AI into core clinical processes. The integration of Artificial Intelligence into Clinical Decision Support Systems (AI-CDSS) highlights a fundamental transformation within healthcare delivery. This transformation enables advanced predictive analytics, multimodal data integration, and real-time augmentation of clinical decisions. However, AI introduces systemic, ethical, operational, and governance risks that challenge traditional healthcare audit and assurance frameworks. In fact, assurance methodologies designed for static software are becoming insufficient for patient safety and regulatory compliance. This review discusses the evolving landscape of AI-CDSS audit, highlighting its transition from a technically focused lifecycle validation to an integrated paradigm centered on socio-technical resilience. Early audit approaches adapted conventional medical device and software validation models to machine learning–enabled clinical tools. While these models established baseline safety oversight, they were not designed to address adaptive algorithms functioning in complex, evolving clinical environments. Modern governance frameworks emphasize continuous performance monitoring, lifecycle surveillance, and structured human oversight. However, a persistent implementation gap remains. Many audit models poorly capture the interactions among algorithmic behavior, clinical workflows, organizational culture, and shifting patient populations. In fact, three major paradigm shifts are reforming the AI-CDSS audit. First, the audit scope is expanding from model-centric evaluation to ecosystem-level assurance that incorporates workflow integration, human-machine collaboration quality, and organizational learning capacity. Second, the field is moving from post-hoc explainability toward reasoning traceability and synergistic clinical sense-making. Third, audit philosophy is shifting from static compliance verification toward resilience-oriented monitoring that focuses on adaptive capacity, graceful degradation, and safe performance evolution. Thus, we propose the STRAICS framework (Socio-Technical Resilience Assurance for Intelligent Clinical Systems), which integrates technical robustness, human-machine interaction safeguards, adaptive governance, and transparency-by-design infrastructure. These components are vital for building trustworthy, effective, and impartial clinical AI ecosystems that can safely manage the increasing complexity of healthcare.

## 1 Introduction

In a short timeframe, Artificial Intelligence (AI) has shifted from a revolutionary concept to an essential part of clinical decision-making. Today, a substantial number of U. S. acute care hospitals have integrated AI-Driven Clinical Decision Support Systems (AI-CDSS) into routine operations, a trend accelerated globally by the shift toward value-based care (Elhaddad and Hamam, 2024; Gomez-Cabello et al., 2024; Sutton et al., 2020). AI is no longer an optional tool; it has become central to modern medicine, from delivering real-time predictive analytics to identify potential sepsis (Mao et al., 2018; Fleuren et al., 2020) to offering expert-level assistance in radiology (Sacoransky et al., 2024; Rockall et al., 2023; Hosny et al., 2018; Langlotz, 2023). Acting as a tireless “co-pilot,” AI enables clinicians to manage overwhelming volumes of patient data, helping them to focus more on direct patient care and less on administrative duties (Olson et al., 2025; Zhao et al., 2025).

However, this rapid adoption has simultaneously revealed a critical and widening assurance gap. Traditional healthcare audit and validation frameworks, designed for static, rule-based software, are incompatible with AI systems that continuously learn, evolve, and generate probabilistic outputs shaped by clinical context and data variation. Conventional validation approaches generally rely on one-time performance verification under controlled conditions, whereas AI-enabled systems require continuous performance monitoring across the product lifecycle to account for model drift, dataset shift, and real-life deployment variability (Sahiner et al., 2023; Kore et al., 2024; Davis et al., 2024; Kim et al., 2025; Subasri et al., 2025; Pennestrì et al., 2025). These issues have prompted regulatory bodies and patient safety researchers to emphasize lifecycle-based oversight, post-deployment surveillance, and risk-based governance frameworks tailored specifically to adaptive machine learning systems in healthcare (Kelly et al., 2019; Dzobo et al., 2020; Wiens et al., 2019; Kokina et al., 2025).

Early approaches to AI-CDSS audit primarily sought to adapt existing medical device and software lifecycle validation models. They stress rigorous pre-deployment testing followed by scheduled post-market surveillance. This phase-gate model assumed relative system stability and clearly bounded operational environments—assumptions increasingly contradicted by empirical evidence (International Medical Device Regulators Forum (IMDRF), 2017; U.S. Food and Drug Administration, 2002, 2019). Mounting reports of post-deployment performance decay, contextual brittleness, and unanticipated failure modes have catalyzed an urgent paradigm transition toward the concept of continuous assurance (Finlayson et al., 2021; Zech et al., 2018; Finlayson et al., 2019). Regulatory entities worldwide have codified this transition toward lifecycle oversight. For instance, the European Union’s Artificial Intelligence Act mandates post-market monitoring, continuous risk management, and real-world performance surveillance for high-risk AI systems, including most clinical applications (Cuocolo et al., 2025; Aboy et al., 2024; EU, 2024). While the United States has not yet enacted legislation formally requiring independent audits of clinical AI systems, several proposed bills and regulatory initiatives point to a trajectory toward accountability. Measures such as the Artificial Intelligence in Health Care Efficiency and Study Act emphasize oversight, transparency, and risk management for high-risk AI systems, complemented by FDA guidance stressing the importance of post-market monitoring and lifecycle performance surveillance (Benjamens et al., 2020; Congressional Research Service, n.d.; Sanal et al., 2019).

Importantly, the technical landscape has experienced a radical transformation. The advancement of healthcare-adapted base models of large-scale, multimodal systems has enabled the processing and synthesis of a variety of data types, including clinical notes, imaging, and genomic sequences. These inventions have rendered many traditional audit approaches obsolete (Moor et al., 2023; Wong et al., 2024; Huang et al., 2025; Ranisch and Haltaufderheide, 2025). Such systems exhibit emergent capabilities and complex failure modes that exceed the bounded logic of conventional software. They function as dynamic collaborators rather than static tools. Beyond performance degradation, AI-CDSS introduce a multidimensional risk landscape which encompasses patient safety, algorithmic bias, cybersecurity, operational disruption, legal liability, regulatory non-compliance, and erosion of clinician trust. Unlike conventional platforms, these risks evolve continuously as AI systems interact with changing clinical environments, healthcare organizations, and human users. Therefore, effective assurance requires not only continuous monitoring of model performance but also continuous identification, assessment, prioritization, and mitigation of risk throughout the AI lifecycle. This risk-oriented perspective provides the conceptual foundation for the STRAICS framework proposed in this review.

This review synthesizes the evolution of AI-CDSS audit, charting its development from technically oriented validation approaches to its current imperative as a socio-technical discipline. The review indicates that effective assurance now calls for a shift beyond lifecycle monitoring toward cultivating socio-technical resilience. To formalize this approach, we introduce the STRAICS Framework (Socio-Technical Resilience Assurance for Intelligent Clinical Systems), which conceptualizes AI-CDSS audit as a continuous, system-level process. STRAICS emphasizes that the integrated system of AI-CDSS and its human, organizational, and procedural contexts must withstand expected stresses, adapt safely, and sustain performance under unforeseen conditions and evolving clinical realities. The framework is structured around three interconnected dimensions: (1) technical robustness, assuring the system maintains reliability in continuous change; (2) explanatory transparency, enabling meaningful human monitoring; and (3) organizational integration, embedding AI safely within care delivery. For each dimension, the review examines its historical foundation, evolution, and forthcoming trajectory within the STRAICS resilience paradigm.

## 2 From discrete lifecycle monitoring to perpetual technical vigilance

The initial wave of AI-CDSS audit strategies largely adapted the phased-gate validation model entrenched in medical device regulation, emphasizing comprehensive pre-market testing followed by periodic post-market surveillance (Koch et al., 2024). Existing regulatory frameworks, such as the traditional medical device lifecycle, rely on a one-time validation and periodic surveillance paradigm, implicitly assuming that a system will remain relatively stable if its operational environment does not change (Rahamtalla et al., 2025; Hellmeier et al., 2024; Matheny et al., 2024). However, empirical evidence now shows that this assumption does not hold for adaptive AI systems deployed in real clinical settings, where model performance may drift or degrade over time and continuous oversight is required to maintain safety and effectiveness (Davis et al., 2024; Raz et al., 2025). Table 1 summarizes key failure modes observed in continuously deployed clinical AI systems, illustrating their definitions, real-world examples, and potential clinical impact.

**Table 1:** Failure mode taxonomy for continuous clinical AI.

Failure mode	Definition	Example in clinical AI	Impact
Dataset drift	Changes in the statistical distribution of input data over time.	Lab values shift due to new instruments or protocols.	Reduced model accuracy; increased misclassification risk.
Concept drift	Changes in the underlying relationship between predictors and outcomes.	Updated treatment guidelines alter outcome correlations.	Model predictions become invalid; may harm clinical decision-making.
Feedback loop bias	Model predictions influence behavior, reinforcing patterns in historical data.	AI predicts sepsis risk → clinicians intervene → new data reinforces model bias.	Amplifies existing biases; may worsen patient outcomes.
Catastrophic forgetting	Loss of previously learned knowledge when retraining on new data.	Continually learning imaging AI forgets rare tumor patterns after retraining.	Previously reliable predictions degrade; patient safety risk.
Automation bias amplification	Overreliance on AI outputs reduces human critical evaluation.	Clinicians follow AI alerts without questioning false positives.	Increases errors; may propagate incorrect diagnoses.

In fact, empirical investigations have shown that clinical machine learning models deployed in real-world healthcare environments frequently experience significant performance deterioration over time, even in the absence of explicit changes to the underlying model code. This degradation is primarily driven by temporal dataset shift and calibration drift, as well as the evolving patient populations. Several studies have shown that clinical prediction models become vulnerable to temporal drift as patient characteristics change across healthcare systems and time periods. Changes in clinical workflows as well as data acquisition practices also make clinical prediction models vulnerable to temporal drift (Sahiner et al., 2023; Rahmani et al., 2023; Lee et al., 2023; Zhang et al., 2022).

Longitudinal analyses of deployed clinical models have documented progressive declines in discrimination metrics. This includes reductions in the area under the receiver operating characteristic curve (AUC), as well as calibration instability. These findings show the inherent delicacy of static validation paradigms when applied to dynamic clinical environments. The findings similarly reinforce the need for continuous performance monitoring, recalibration, and model updating throughout the operational lifecycle of clinical AI systems (Subasri et al., 2025; Lee et al., 2023; Zhang et al., 2022). Mechanistically, the performance decay comes from multiple interacting forms of drift. Data drift occurs when the statistical distribution of input variables changes, for example, due to new laboratory instrumentation. It can also occur due to updated diagnostic workflows or shifts in patient demographics. In contrast, concept drift reflects changes in the underlying relationship between predictors and clinical outcomes. This drift is often prompted by evolving treatment standards, new therapeutic interventions, or redefined disease classifications. Together, these processes fundamentally challenge the assumption that a model validated at deployment will maintain stable performance over time (Sahiner et al., 2023). The ramifications for assurance and governance are substantial. Evidence from clinical AI monitoring research shows that models left unmonitored in production environments can silently degrade, potentially increasing the risk of clinical misclassification and patient harm. As a result, there is a growing consensus that clinical AI systems require uninterrupted monitoring infrastructures capable of detecting drift to trigger recalibration or retraining, and to document performance changes for regulatory and safety oversight (Rahmani et al., 2023; Zhang et al., 2022). Early regulatory responses, such as the FDA’s Artificial Intelligence/Machine Learning Software as a Medical Device Action Plan, introduced the concept of Predetermined Change Control Plans (PCCPs) to allow manufacturers to prospectively define the scope, methodology, and validation strategy for anticipated algorithm modifications (U.S. Food and Drug Administration, 2021). PCCPs were intended to enable safe iterative updates while maintaining regulatory oversight and assurance of device safety and effectiveness across the product lifecycle (U.S. Food and Drug Administration, 2023, 2024). However, while these frameworks addressed planned and bounded algorithmic evolution, they struggled to fully accommodate the unpredictable, multifactorial nature of real-world concept drift and evolving clinical practice environments.

**Figure 1:**
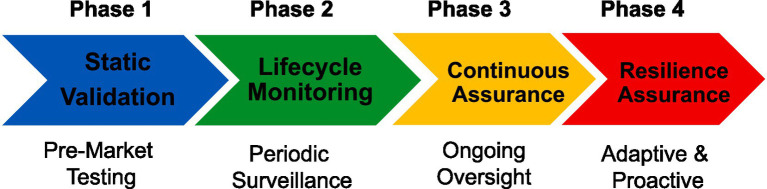
Evolution of Clinical AI Assurance. This figure illustrates the progression of audit and validation strategies for AI-driven clinical decision support systems (AI-CDSS) from static pre-market validation to lifecycle monitoring, continuous assurance, and finally, resilience-oriented assurance. Each phase reflects increasing levels of oversight, adaptability, and integration into dynamic clinical environments.

Interestingly, the technical paradigm has simultaneously shifted toward continuously learning and distributed training architectures capable of updating model parameters using real-world clinical data streams. Privacy-preserving approaches such as federated learning and swarm-like distributed learning frameworks have emerged as key enablers of this transition, allowing multiple institutions to collaboratively train models without centralizing sensitive patient data while supporting applications across medical imaging, oncology, infectious disease modeling, and precision medicine (Miloudi et al., 2026; Warnat-Herresthal et al., 2021; Sandhu et al., 2023). Systematic reviews have demonstrated rapid expansion of federated learning across clinical domains, driven by privacy requirements, multi-institutional data disparity, and the need for scalable model generalization in real-world care environments. These developments have further complicated traditional audit and validation paradigms by introducing distributed model governance, asynchronous parameter updates, and heterogeneous data ecosystems. As a result, assurance strategies must shift from episodic validation toward continuous performance monitoring and adaptive lifecycle governance capable of operating across decentralized model training environments (Teo et al., 2024). The progression from static validation to resilience-oriented assurance is illustrated in Figure 1.

While this paradigm directly addresses dataset drift through ongoing adaptation, it introduces a new generation of audit risks that require continuous technical oversight or perpetual technical vigilance. One such risk is catastrophic forgetting, in which sequential model retraining on new data can degrade performance on previously learned clinical tasks. This phenomenon has been specifically documented as a major barrier to real-world deployment of continually learning medical imaging models across hospitals and evolving patient populations (Leonard and Hidayaturrahman, 2025). Additional risks include feedback loop bias, in which models trained on historical clinical decisions may reinforce existing diagnostic or treatment patterns, potentially amplifying systematic biases and inequities in care delivery. Concerns regarding algorithmic bias and performance disparities across patient subpopulations have been widely documented in real-world healthcare machine learning deployments, which highlights the need for fairness-aware monitoring and governance (Huang et al., 2024). Lastly, distributed training environments introduce version proliferation risks, whereby local model adaptation across institutions produces heterogeneous model variants. This complicates centralized oversight, traceability, and accountability. These realities collectively reinforce the need for continuous, risk-based, and socio-technical assurance frameworks capable of maintaining safety and performance across dynamic, multi-institutional AI ecosystems. To address these challenges systematically, we propose the STRAICS Framework, which structures resilience-oriented audit around continuous technical oversight, explanatory transparency, and organizational integration across dynamic AI-CDSS environments. To operationalize continuous assurance, retraining triggers must be precisely defined. We propose multi-metric thresholds grounded in statistical process control: Population Stability Index exceeding 0.1 warrants investigation, while exceeding 0.2 triggers retraining. Area under the ROC Curve degradation exceeding 5%, calibration slope deviation exceeding 20%, and Brier score increase exceeding 15% each mandate review. Temporal triggers recommend retraining every 6–12 months with a minimum of 5,000 patient encounters. Composite weighted scoring prevents false alarms. A three-tier alert system categorizes severity: yellow (investigate), orange (recalibrate), and red (rollback) (Table 2). These specifications align with FDA Predetermined Change Control Plan principles (U.S. Food and Drug Administration, 2021, 2024) and ISO/IEC TR 24028 trustworthiness frameworks (ISO/IEC TR 24028, 2020). However, institutions must calibrate thresholds to their clinical risk tolerance and patient populations.

**Table 2:** Model retraining trigger decision cascade.

Step	Action	Trigger/criteria
1	Continuous monitoring	AUC, calibration slope, PSI, Brier Score tracked in real-time
2	Threshold check	PSI > 0.1; AUC drop >5%; Cal deviation >20%; Brier >15%; Time >6 months
3	Alert assignment	Yellow (investigate); orange (recalibrate); red (rollback)
4	Human review	Clinical impact evaluation; root cause analysis
5	Decision	Continue monitoring; recalibrate; rollback to previous version
6	Documentation	Incident logging; performance records; learning loop

Contemporary technical audit must, therefore, extend far beyond the periodic verification of performance metrics against a static baseline. It must evolve to assess the governance of the learning process itself. Within the STRAICS paradigm, technical audit encompasses not only continuous monitoring of model performance but also the proactive assessment of adaptive learning processes, risk mitigation strategies, and system-level resilience under evolving clinical conditions. Recent international standards, such as ISO/IEC TR 24028 on trustworthiness in artificial intelligence, provide structured taxonomies for evaluating robustness, reliability, resilience, safety, transparency, and accountability across the full AI lifecycle, reinforcing the need for continuous monitoring and risk-based assurance approaches in high-stakes domains such as healthcare (ISO/IEC TR 24028, 2020; ISO 24028 Artificial Intelligence – Trustworthiness, 2020.). In parallel, regulatory science has increasingly shifted toward lifecycle-based oversight and operational monitoring rather than static pre-deployment validation. Although not originally designed specifically for continuously learning clinical AI, recent FDA pilot programs and internal AI deployment initiatives highlight a broader institutional transition toward operational AI governance, automation of monitoring workflows, and integration of AI into regulatory decision-support infrastructure (FDA Announces Completion of First AI-Assisted Scientific Review Pilot and Aggressive Agency-Wide AI Rollout Timeline, 2025).

This transition reflects a broader maturation from simple detection of performance deviations toward managed response paradigms, in which drift, anomalies, and safety signals trigger predefined technical and governance actions. Emerging audit concepts are beginning to extend beyond output monitoring toward evaluation of internal model behavior, knowledge representation, and decision logic stability over time—an evolution consistent with trustworthiness frameworks emphasizing explainability, controllability, and accountability across deployment environments (ISO 24028 Artificial Intelligence – Trustworthiness, 2020). The future trajectory increasingly points toward resilience-oriented technical audit, focused on adaptive capacity, graceful degradation under stress, and validated recovery mechanisms following exposure to anomalous or shifted data distributions.

## 3 From post-hoc explainability to real-time reasoning assurance

The imperative for transparency in AI-CDSS emerged early, driven by ethical considerations and the need for clinical trust. Initial strategies heavily emphasized post-hoc explainability techniques, such as SHapley Additive exPlanations (SHAP) and Local Interpretable Model-agnostic Explanations (LIME), which generate feature importance scores or local approximations to explain individual predictions after they are made (Ribeiro et al., 2016; Lundberg and Lee, 2017). However, a substantial and growing body of evidence demonstrated that these post-hoc explanations can be misleading, unstable across similar inputs, or clinically implausible when applied to complex medical models (Ghassemi et al., 2021; Rudin, 2019). These methods risk creating an illusion of interpretability—providing the appearance of transparency while leaving underlying model behavior poorly understood. This precipitated a critical audit dilemma: should regulatory and institutional compliance be satisfied by the mere presence of an explanation interface, or must the explanations themselves be validated for accuracy, stability, and clinical plausibility (Ghassemi et al., 2021; Doshi-Velez and Kim, 2017)? This period was characterized by intense debate between advocates of interpretability-by-design, who argued for inherently transparent models even at potential cost to predictive performance (Rudin, 2019), and proponents of high-performance complex models validated through rigorous external and prospective testing, who viewed post-hoc explanations as supportive but not foundational to safety or clinical reliability (Rajkomar et al., 2019; Topol, 2019).

The emergence of large-scale foundation models in healthcare has reshaped the transparency and auditability landscape. Earlier predictive clinical models produced bounded outputs such as binary classifications or risk probabilities. However, contemporary clinical foundation models generate high-dimensional outputs, including structured clinical narratives and ranked differential diagnoses. This is in addition to longitudinal management strategies. These generative capabilities introduce new epistemic and governance challenges because traditional explainability approaches are often computationally impractical and clinically insufficient for interpreting complex reasoning processes. As demonstrated in large clinical language model studies, performance improvements are increasingly linked to emergent reasoning behaviors rather than isolated feature-output mappings (Singhal et al., 2023).

For generative and reasoning-centric clinical AI systems, conventional explainability paradigms therefore provide limited assurance value. Highlighting token-level or pixel-level importance fails to capture the hierarchical inference structure underlying diagnostic reasoning and treatment prioritization. Thus, the audit paradigm is shifting from static explainability toward reasoning traceability, the capacity to reconstruct and evaluate the sequential inferential pathway that links inputs, intermediate representations, and final recommendations. Recent work in clinical machine learning governance emphasizes that safe deployment increasingly depends on the ability to audit reasoning consistency, counterfactual stability, and uncertainty calibration across clinical contexts rather than merely inspecting feature attribution maps (Ghassemi et al., 2021).

To this shift into operation, emerging evaluation ecosystems are focusing on multidimensional reasoning quality rather than single-metric performance. Benchmarking initiatives such as MedExplain-style evaluation frameworks, developed through multi-institutional collaborations, have proposed standardized assessment across domains including factual accuracy, clinical actionability, causal plausibility, uncertainty quantification, and counterfactual reasoning robustness (Rajpurkar et al., 2022). These structured evaluation approaches allow auditors and regulators to move beyond model accuracy toward clinically meaningful trustworthiness metrics. Importantly, these developments are beginning to influence regulatory and accreditation expectations. Healthcare oversight bodies are increasingly incorporating AI governance requirements, emphasizing transparency documentation. Model validation traceability, and post-deployment performance monitoring are also emphasized. Recent accreditation and safety guidance documents highlight the need for auditable AI lifecycle controls, including evidence of model reasoning reliability and documented evaluation across representative patient populations (Joint Commission and Coalition for Health AI, 2025). Together, these trends signal a transition toward audit-ready AI, where models must not only perform well but also demonstrate reconstructable and clinically defensible reasoning pathways throughout their operational lifecycle.

Furthermore, contemporary AI-CDSS audit must address the critical dimension of human–AI collaborative reasoning quality. As clinical AI systems increasingly influence high-stakes diagnostic and therapeutic decisions, accountability frameworks are shifting toward shared responsibility models spanning developers, healthcare institutions, and end-user clinicians. Emerging medico-legal scholarship and regulatory commentary have emphasized that inappropriate delegation of clinical judgment to automated systems may expose both manufacturers and healthcare organizations to liability, particularly when human oversight processes are poorly defined or inadequately implemented (Topol, 2019; Price 2nd and Cohen, 2019). High-profile medico-legal analyses published in leading clinical journals have further reinforced that clinicians retain ultimate responsibility for patient-level decisions, even when supported by advanced AI recommendations (Topol, 2019; Price and Cohen, 2019). This evolving legal and ethical landscape creates new, non-negotiable audit imperatives. Audit frameworks must now verify the existence, clarity, and operationalization of shared decision-making protocols that delineate clinical scenarios in which AI outputs may inform autonomous action versus those requiring mandatory human confirmation, escalation, or override (U.S. Food and Drug Administration, 2021). In parallel, regulators and patient safety organizations increasingly emphasize human factors–aware AI governance, requiring evaluation not only of model performance but also of how outputs are presented within clinical workflows and cognitive environments (Sendak et al., 2020). Poor interface design or overconfident probability presentation may amplify automation bias, anchoring effects, or cognitive overload, thereby degrading clinical decision quality even when underlying model performance is technically sound (Ghassemi et al., 2021; Sendak et al., 2020). In this context, systematic analysis of clinician interaction patterns, including override frequency, response latency, and disagreement clustering, has emerged as a critical source of audit intelligence. Such signals can reveal latent model errors, usability friction, or early evidence of dataset or concept drift before degradation becomes detectable through conventional performance metrics (Finlayson et al., 2019; Davis et al., 2019). Therefore, contemporary assurance strategies increasingly conceptualize safety as an emergent property of the joint cognitive system formed by clinicians, AI tools, and organizational workflows, rather than as a characteristic of the algorithm in isolation (Amann et al., 2020). This shift marks a maturation of audit philosophy from static technical validation toward dynamic socio-technical performance assurance across real-world care environments.

## 4 From organizational compliance to socio-technical resilience

The initial implementation phase of AI-CDSS revealed a consistent pattern represented by a strong technical performance in controlled, retrospective validation studies. However, this performance often failed to translate into sustained clinical impact in real-world care environments. This translational gap reflects the inherent complexity of healthcare delivery. Institutional constraints, workflow variability, and patient heterogeneity dynamically interact with algorithmic outputs (Sendak et al., 2020). In fact, implementation science research has demonstrated that the clinical effectiveness of systems such as sepsis early warning models depends more on socio-technical integration and less on discrimination metrics. In other words, the clinical effectiveness of systems is dependent on how alerts are embedded within the providers’ workflows, the clarity and operational feasibility of escalation pathways, alignment with institutional resources, and the timing and cognitive burden of notifications (Wong et al., 2021). This phenomenon, often described as the “last-mile problem” in clinical AI, underscores that AI-CDSS safety and effectiveness are not intrinsic properties of the model itself but rather emergent properties of the broader clinical ecosystem in which the technology is deployed (Sendak et al., 2020; Amann et al., 2020). Early real-world deployment studies demonstrated that poorly integrated decision support tools may increase alert fatigue, workflow fragmentation, or automation bias, even when underlying predictive performance remains technically robust (Sutton et al., 2020; Wong et al., 2021).

Early organizational audit responses frequently focused on verifying the existence of formal governance infrastructure, such as AI oversight committees, clinician education programs, and incident reporting mechanisms. However, subsequent health informatics and safety science research has shown that structural presence alone does not guarantee functional risk mitigation. Checklist-driven governance approaches often document procedural compliance without evaluating whether governance processes actively detect model degradation, usability failures, or unintended clinical consequences during real-world use (Cutillo et al., 2020; Bates and Singh, 2018). Accordingly, contemporary organizational AI audit frameworks are shifting toward functional governance assurance, emphasizing real-time monitoring of clinical workflow integration, continuous evaluation of the quality of human-AI interaction, and outcome-linked governance performance metrics. This evolution reflects a broader transition in healthcare safety science from structural compliance toward adaptive system resilience and learning health system principles (Friedman et al., 2017).

The healthcare ecosystem itself has been transforming in ways that deepen socio-technical complexity. The ongoing economic transition toward value-based care and alternative payment models has increasingly linked reimbursement to longitudinal outcomes as well as care coordination quality and continuous performance measurement rather than episodic encounters. Recent updates to the CY 2026 Medicare Physician Fee Schedule (PFS) reinforce this shift by emphasizing longitudinal care delivery models, remote monitoring integration, and operational accountability for continuous patient management workflows. Such emphasis indirectly incentivizes sustained monitoring and documentation of technology-enabled care processes rather than one-time validation snapshots (Calendar Year, 2026; CMS releases CY 2026 Physician Fee Schedule Final Rule, 2026). At the same time, the physical and operational footprint of AI within healthcare delivery has expanded dramatically. AI capabilities are now embedded directly into smart medical devices, including robotic surgical platforms, intelligent infusion systems, and remote monitoring ecosystems. They are also increasingly ambient through voice-enabled clinical documentation, environmental sensors, and continuous physiologic monitoring infrastructures. This expansion exponentially increases the technical audit surface area. It requires assurance not only of algorithmic performance but also of device security, sensor reliability, and network integrity across distributed clinical environments.

These needs align with evolving cybersecurity governance frameworks such as HITRUST CSF v11.2, which was updated to better address emerging threat landscapes, streamline overlapping controls, and maintain alignment with evolving regulatory and security standards (HITRUST Updates October 2023, 2023; HITRUST. 9 practical benefits of HITRUST Certification, 2026). The HITRUST framework itself integrates controls across dozens of regulatory and industry standards and has increasingly incorporated AI-relevant risk domains and certification pathways as healthcare systems adopt more autonomous and data-intensive technologies (HITRUST, 2026). Together, these developments are transforming audit from a narrow safety and compliance function into a foundational component of enterprise risk management, revenue integrity, and operational resilience in AI-enabled healthcare delivery.

In response to these layered complexities, we propose that AI-CDSS audit must evolve toward a socio-technical resilience assurance framework. We formalize this approach as the STRAICS Framework, which operationalizes resilience-oriented audit by integrating technical, clinical, organizational, and regulatory-economic dimensions into a unified, adaptive assessment model. Distinct from existing resilience engineering, learning health system, and AI governance frameworks, STRAICS is proposed as an operational assurance framework specifically for adaptive AI-driven clinical decision support systems. While it draws on resilience engineering concepts such as anticipation, monitoring, response, and learning, STRAICS translates these principles into auditable AI-specific functions, including model drift surveillance, human-AI oversight mechanisms, equity monitoring, lifecycle governance, and regulatory-economic adaptation. Likewise, although learning health systems emphasize continuous organizational learning, they do not explicitly address the unique assurance challenges posed by continuously evolving machine-learning models. The contribution of STRAICS, therefore, lies in integrating AI lifecycle management, socio-technical resilience, human-AI collaboration, and regulatory oversight into a unified framework for continuous assurance of AI-CDSS. Rather than focusing narrowly on static compliance or episodic performance verification, a resilience-oriented audit evaluates the capacity of AI-enabled clinical systems to maintain safe and effective operation under conditions of uncertainty, operational stress, and continuous change. This perspective is grounded in resilience engineering and safety science literature, which conceptualize safety as the ability of complex systems to adapt, anticipate, monitor, and respond to variability rather than simply prevent isolated failures (Braithwaite et al., 2015; Hollnagel, 2014). Table 3 contrasts traditional audit practices with the STRAICS approach.

**Table 3:** Traditional audit vs. resilience audit.

Aspect	Traditional audit	Resilience-oriented audit (STRAICS)
Focus	Compliance with documented procedures and static policies	System performance, adaptation, and learning under real-world conditions
Validation approach	One-time pre-deployment testing; periodic post-market checks	Continuous lifecycle monitoring, including real-time drift detection and recovery capability
Integration	Evaluates artifact existence (policies, committees, SOPs)	Assesses socio-technical system integration (human-AI collaboration, workflow alignment)
Performance metrics	Accuracy, sensitivity, and specificity at a single time point	Adaptive performance measures, including graceful degradation, recovery velocity, and equity surveillance
Governance	Checklist-driven, procedural compliance	Dynamic, cross-functional oversight; continuous feedback loops and learning cycles
Risk management	Reactive; focused on known hazards	Proactive and adaptive; anticipates drift, failure modes, and emergent risks
Outcome orientation	Episodic reporting for regulatory documentation	Continuous assurance of safety, effectiveness, and resilience in dynamic clinical environments

**Figure 2:**
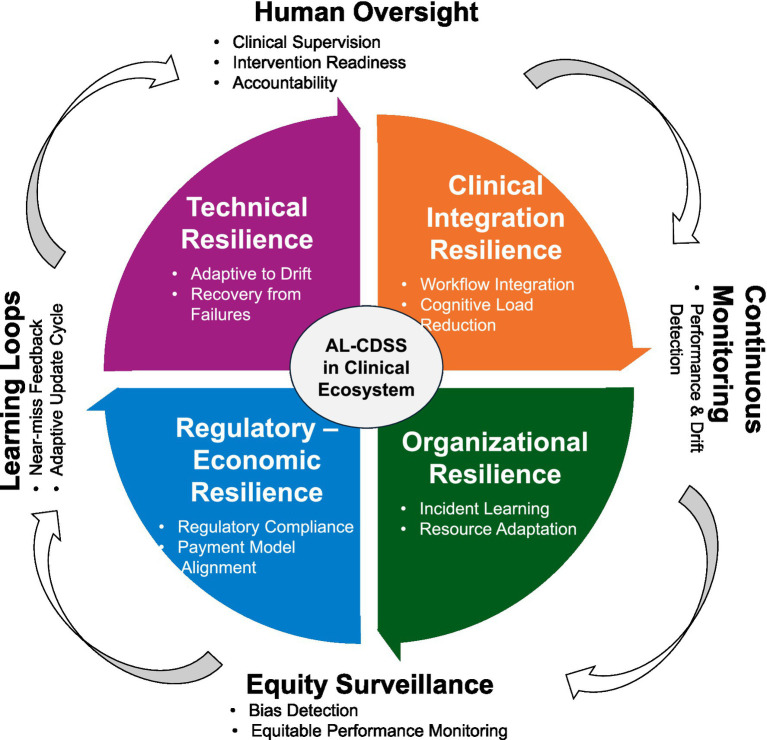
STRAICS Framework. This figure illustrates the AI-CDSS at the center of a clinical ecosystem, emphasizing resilience-oriented assurance across four interconnected domains: Technical resilience: system adaptability to dataset and concept drift, robustness to failure, and capacity for recovery after degradation events; Clinical integration resilience: alignment with clinical workflows, optimization of cognitive load, and support for effective human-AI collaboration; Organizational resilience: institutional capabilities for incident learning, adaptive resource allocation, and continuous improvement; Regulatory–economic resilience: compliance with evolving regulations, alignment with performance-linked reimbursement models, and sustainability of AI-CDSS implementation. Outer ring (cross-cutting mechanisms): Continuous monitoring: ongoing observation of system performance and drift detection; Human oversight: active clinical supervision, intervention capability, and accountability in decision-making; Learning loops: feedback-driven adaptation from near-misses, adverse events, and workflow observations; and equity surveillance: detection and mitigation of biases, ensuring fair and equitable system performance across patient populations.

Within this paradigm, AI-CDSS implementations should be evaluated across four interconnected domains: technical resilience, clinical integration resilience, organizational resilience, and regulatory-economic resilience (Figure 2). Technical resilience encompasses the system’s adaptive capacity, robustness to dataset and concept drift, failure mode characteristics, and recovery mechanisms following degradation events. Contemporary AI governance literature emphasizes that continuously learning and adaptive machine learning systems must be evaluated across their full operational lifecycle rather than through one-time validation checkpoints (EU, 2024; U.S. Food and Drug Administration, 2021). Clinical integration resilience evaluates how AI systems interact with real clinical workflows, including their effects on cognitive load, automation bias, and human-AI collaborative decision quality. Implementation science and human-factors research demonstrate that clinical impact depends heavily on workflow alignment, alert timing, usability, and the degree to which decision support augments rather than disrupts clinician cognition (Ghassemi et al., 2021; Sendak et al., 2020). Organizational resilience examines institutional capacity to detect, learn from, and adapt to system anomalies. Learning health system frameworks emphasize continuous feedback loops, incident learning, adaptive resource allocation, and clear accountability structures as prerequisites for safe deployment of data-driven clinical technologies (Bates and Singh, 2018; Friedman et al., 2017). Lastly, regulatory-economic resilience assesses the sustainability of AI-CDSS implementation across evolving regulatory environments and payment structures. As healthcare financing models increasingly reward longitudinal outcomes, quality metrics, and population-level performance, organizations must ensure that AI governance structures remain adaptable to shifting compliance expectations and performance-linked reimbursement models (Committee on Accounting for Socioeconomic Status in Medicare Payment Programs, 2016; QPP, 2026). Figure 2 illustrates the STRAICS Framework, showing AI-CDSS embedded in the clinical ecosystem with four resilience domains and an outer ring of governance and monitoring mechanisms critical for safe and equitable operation.

Under this framework, audit moves beyond verifying whether governance artifacts exist (i.e., asking “Is the policy documented?”) to evaluating how the integrated socio-technical system, technology, clinicians, and organizational processes, adapts, learns, and maintains safety under real-world variability (i.e., asking “How does the entire system adapt, learn, and maintain safety when faced with unexpected pressures or inevitable change?”). Therefore, audit methodologies must evolve to incorporate system stress-testing through simulation and direct observation of real-world clinical use. Structured analysis of organizational learning cycles derived from near-misses and adverse events must also be incorporated. These approaches align with modern patient safety science, which emphasizes proactive risk identification, adaptive monitoring, and continuous system learning (Bates and Singh, 2018; Hollnagel, 2014; Committee on Accounting for Socioeconomic Status in Medicare Payment Programs, 2016).

## 5 Regulatory convergence and persistent implementation gaps

The regulatory landscape for artificial intelligence in healthcare has evolved from fragmented, principle-based guidance toward increasingly structured, enforceable, and internationally harmonized governance frameworks. The European Union’s Artificial Intelligence Act establishes a legally binding, risk-based regulatory structure for AI systems, including mandatory classification of high-risk applications, lifecycle risk management requirements, post-market monitoring obligations, and enforceable human oversight provisions (EU, 2024). In parallel, the United States has advanced a layered regulatory strategy combining sector-specific oversight, agency guidance, and legislative proposals aimed at strengthening transparency, safety evaluation, and accountability for high-risk AI systems used in healthcare delivery (The White House, 2023; AI Risk Management Framework, 2023).

Although the United States has not yet enacted a single comprehensive healthcare-specific AI statute equivalent to the EU AI Act, recent legislative proposals and federal policy frameworks converge on similar core governance principles. These include risk stratification of AI systems, lifecycle performance monitoring, transparency documentation, and explicit requirements for human-in-the-loop accountability for clinical decisions (The White House, 2023; AI Risk Management Framework, 2023). Regulatory agencies such as the FDA have reinforced this trajectory through lifecycle-oriented regulatory models for adaptive machine-learning software as a medical device, including expectations for post-deployment performance monitoring and controlled pathways for model modification (U.S. Food and Drug Administration, 2021). At the international level, standards organizations have contributed to foundational technical frameworks that facilitate regulatory convergence. ISO/IEC 23894 provides a structured risk management framework for AI systems, establishing common terminology, risk identification methodologies, lifecycle risk monitoring approaches, and governance integration principles that can be applied across jurisdictions and regulatory regimes (International Organization for Standardization, 2023). These standards are increasingly used to translate regulatory expectations into implementable organizational controls and audit methodologies. Together, this growing convergence across legislation, regulatory guidance, and international technical standards provides a more stable and predictable foundation for the development of scalable AI audit protocols. Such harmonization is particularly critical for multinational healthcare organizations that must demonstrate consistent AI governance, performance monitoring, and safety assurance across multiple regulatory environments while maintaining operational efficiency and clinical effectiveness (Hellmeier et al., 2024; U.S. Food and Drug Administration, 2021; International Organization for Standardization, 2023). Nevertheless, despite this regulatory convergence, translating risk-based governance principles into operational practice remains challenging. Effective AI assurance requires continuous management of interconnected technical, clinical, organizational, regulatory, and ethical risks throughout the AI lifecycle. Thus, risk assessment should not be viewed as a one-time pre-deployment exercise but as a continuous process of identification, evaluation, mitigation, and reassessment as AI systems evolve in real-world clinical environments. This perspective aligns with the resilience-oriented philosophy of the STRAICS framework and complements emerging lifecycle-based regulatory approaches.

Nevertheless, significant gaps persist between regulatory theory and audit practice. A fundamental temporal mismatch exists, whereby regulatory development and revision cycles, often measured in years, struggle to keep pace with AI development and deployment cycles, which frequently occur over months or even weeks. This regulatory lag is particularly problematic for adaptive and continuous learning systems, where performance, risk profile, and clinical impact can evolve rapidly after deployment. At the same time, healthcare institutions operating across jurisdictions must reconcile divergent regulatory expectations, creating operational complexity for multinational health systems and digital health vendors. Most critically, granular guidance remains limited regarding what constitutes sufficient evidence of safety and effectiveness for continuously learning, non-deterministic systems. Evidence frameworks designed for static imaging algorithms or locked software models are poorly suited to foundation models that generate clinical narratives, care plans, and probabilistic reasoning outputs that evolve over time.

In parallel, structural inequities in healthcare resources further complicate regulatory implementation. Emerging evidence suggests that AI adoption and monitoring capacity are unevenly distributed across healthcare systems, with lower adoption rates in socioeconomically deprived regions and rural healthcare settings. These disparities risk amplifying pre-existing differences in care quality and outcomes if advanced monitoring, validation infrastructure, and specialized governance expertise remain concentrated in well-resourced academic centers (Hwang et al., 2026). Furthermore, systematic analyses of AI deployment in primary care and other clinical domains demonstrate that AI systems can both mitigate and exacerbate health inequities depending on implementation context, data representativeness, and governance maturity (d'Elia et al., 2022). Overall, these realities raise concern that current regulatory and audit frameworks, often designed assuming access to specialized personnel, advanced monitoring infrastructure, and legal resources, may inadvertently create a two-tiered ecosystem of AI assurance, reinforcing rather than reducing global and regional health disparities.

## 6 Future directions: cultivating resilient clinical AI ecosystems

Looking forward, the field must prioritize research that directly bridges the persistent gap between regulatory theory and operational audit practice while putting into operation a resilience-oriented assurance paradigm. We formalize this approach as STRAICS, providing a structured framework to guide adaptive audit, continuous monitoring, and lifecycle-based assurance of AI-enabled clinical systems. A critical priority is the development of adaptive audit methodologies capable of dynamically adjusting scrutiny intensity based on real-time risk signals, mirroring the shift toward lifecycle-based oversight already emerging in regulatory science for AI-enabled medical technologies. Regulatory bodies increasingly emphasize continuous post-market performance monitoring, real-world evidence integration, and structured change management frameworks for adaptive algorithms. This signals a transition away from static, point-in-time validation models toward continuous assurance ecosystems (U.S. Food and Drug Administration, 2021, 2023, 2024). Trigger thresholds require institutional calibration based on clinical risk tolerance, patient population characteristics, and validation resources.

Future research should also focus on developing standardized methodologies for AI risk assessment capable of integrating technical, clinical, organizational, ethical, and regulatory risks into unified assurance models. Such approaches could support dynamic risk scoring, adaptive audit intensity, and prioritization of monitoring resources according to changing clinical conditions and institutional risk tolerance. Developing validated AI risk taxonomies and methods for measuring residual risk following mitigation strategies represents another important research priority. Recent conceptual frameworks have likewise emphasized adaptive, risk-aware governance as a cornerstone of trustworthy and resilient AI assurance, reinforcing the direction proposed by the STRAICS framework (Kandikatla and Radeljic, 2025a,b).

**Figure 3:**
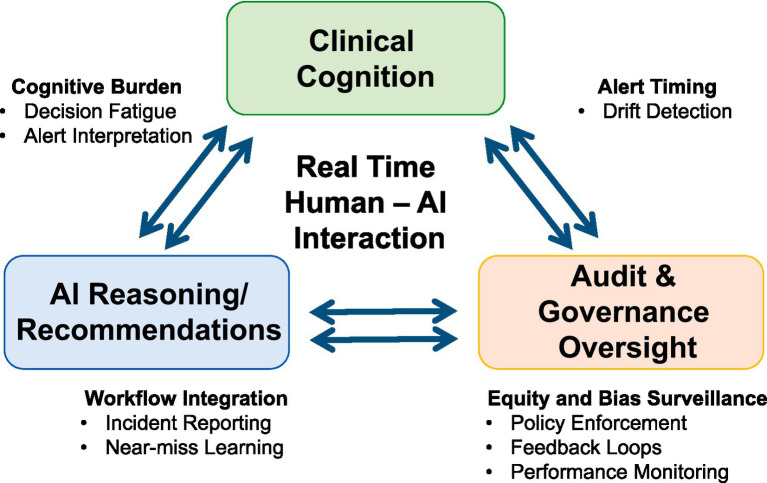
Human–AI joint cognitive safety model.

Parallel to methodological evolution, there is an urgent need to develop validated, standardized metrics for socio-technical resilience. Current performance measures, such as discrimination or calibration metrics, are insufficient to characterize system safety in adaptive environments. Emerging regulatory and standards frameworks increasingly emphasize lifecycle risk management, continuous quality monitoring, and system-level safety evaluation, which could serve as scaffolding for resilience metrics capturing adaptive capacity, recovery velocity following performance perturbations, and institutional learning effectiveness following near-misses or adverse events (Sendak et al., 2020; International Organization for Standardization, 2023). Another major research priority is establishing evidence-based models for optimal human-AI collaborative reasoning across varying clinical contexts, workload conditions, and cognitive environments. Increasing evidence demonstrates that AI effectiveness is tightly coupled to workflow integration, interface design, and clinician cognitive burden rather than algorithmic performance alone. This reinforces the need for audit frameworks that evaluate joint human-machine system behavior rather than isolated model performance (McCradden et al., 2020). Figure 3 illustrates the Human–AI Joint Cognitive Safety Model, depicting real-time feedback loops between clinician cognition, AI reasoning, and governance oversight to support safe and effective clinical decision-making.

Regulatory innovation is equally critical. Emerging regulatory science explores adaptive oversight mechanisms, including structured modification protocols and expanded use of real-world evidence. Lifecycle monitoring requirements are also being explored because they collectively approximate concepts of adaptive licensing, controlled deployment environments, and outcome-linked reimbursement models (U.S. Food and Drug Administration, 2021; U.S. Food and Drug Administration, 2024; U.S. Food and Drug Administration, 2021). These approaches incentivize continuous safety validation and real-world effectiveness rather than one-time compliance demonstration. Lastly, an equity-centered audit paradigm is essential. Continuous learning systems trained using real-world clinical data risk amplifying historical structural inequities embedded in healthcare delivery patterns. Without proactive bias surveillance, fairness auditing, and population-specific performance monitoring, adaptive models may worsen outcome disparities across demographic or socioeconomic groups. This risk is particularly pronounced in resource-limited healthcare settings, where institutions may lack the infrastructure required for continuous monitoring, creating the potential for a two-tiered AI safety ecosystem (McCradden et al., 2020).

Addressing implementation barriers in resource-constrained settings is essential. We propose tiered pathways aligned with institutional resources: Level 1 (minimal adoption) implements basic drift detection using open-source toolkits and manual audits; Level 2 (integrated monitoring) establishes automated performance surveillance and dedicated governance; Level 3 (full STRAICS) implements continuous monitoring, equity surveillance, and adaptive governance. For resource-limited settings, cloud-based shared services, open-source libraries, and federated benchmarking consortia can lower barriers. However, differential implementation capacity may widen disparities if advanced monitoring remains concentrated in well-resourced centers. Implementation science research must evaluate STRAICS feasibility, cost-effectiveness, and unintended consequences across safety-net hospitals, rural systems, and international settings with limited infrastructure.

For healthcare organizations navigating this complex transition, practical pathways can be clearly defined. Establishing integrated, cross-functional AI governance structures with genuine authority and diverse representation is a foundational step. Table 4 presents a maturity model for hospitals implementing AI-CDSS, illustrating the progression from minimal adoption toward full STRAICS-based resilience assurance.

**Table 4:** Implementation maturity model for hospitals.

Level	Adoption stage	Key characteristics	Clinical and organizational focus	Resource requirements
Level 1	Minimal adoption	Checklist compliance; static policies; episodic audits	Basic awareness of AI systems; limited integration into workflows; minimal monitoring	Minimal: basic IT support; manual drift checks; standard EHR reporting tools
Level 2	Integrated monitoring	Partial workflow integration; lifecycle monitoring of AI performance; some human-AI collaboration	Proactive identification of drift, partial feedback loops, and early human oversight mechanisms	Moderate: dedicated data science support; monitoring software; QA team; governance committee
Level 3	Full STRAICS adoption	Adaptive resilience; continuous monitoring; learning loops; equity surveillance; full socio-technical integration	Real-time performance and bias monitoring; dynamic workflow alignment; institutional learning and adaptive governance; continuous risk mitigation	Substantial: AI operations team; real-time monitoring infrastructure; automated alerting systems; data engineering; compliance personnel

Effective governance models emphasize interdisciplinary collaboration among clinicians, data scientists, legal experts, and compliance professionals to manage the complexity and risk profile of clinical AI deployment (Hayyatic AI, 2025). Such cross-functional structures improve accountability, regulatory alignment, and lifecycle oversight, particularly as AI systems evolve after deployment (Hayyatic AI, 2025). Proactively conducting resilience stress testing can reveal latent vulnerabilities before they manifest as patient safety events or operational disruptions. Such testing includes simulation of rapid performance decay, cyber-intrusion, data pipeline failure, or sudden regulatory change. Furthermore, continuous monitoring, anomaly detection, and lifecycle risk management are increasingly recognized as essential components of AI system assurance and incident response readiness in healthcare environments (ClearDATA, 2026). Investment in transparency-by-design infrastructure is a technical prerequisite for future-proof assurance. Modern governance and cybersecurity guidance increasingly emphasize immutable audit trails, detailed activity logging, and continuous monitoring of AI workloads to support regulatory compliance, forensic analysis, and real-time risk detection (ClearDATA, 2026). These capabilities also support emerging expectations for explainability, accountability, and traceability in high-risk AI deployments, particularly in clinical decision support contexts (Project Management Institute, 2025). To illustrate practical implementation, consider a sepsis early warning system deployed within a hospital network (Mao et al., 2018; Fleuren et al., 2020; Rahmani et al., 2023; Wong et al., 2021). Under the STRAICS framework, continuous assurance would extend beyond initial validation to include ongoing monitoring of predictive performance, alert burden, false-positive rates, and evidence of dataset shift resulting from changing patient populations or clinical workflows. When predefined performance thresholds are exceeded, escalation protocols could trigger model review, recalibration, or temporary rollback to a previously validated version while maintaining clinician oversight. Similarly, in medical imaging AI applications, continuous assurance would include surveillance for changes in image acquisition protocols, scanner upgrades, or demographic shifts that may affect model performance (Sacoransky et al., 2024; Rockall et al., 2023; Hosny et al., 2018; Kore et al., 2024; Koch et al., 2024). Human-AI oversight mechanisms would monitor disagreement rates between radiologists and AI recommendations, while anomaly detection systems could identify unexpected degradation requiring investigation. These examples demonstrate how STRAICS complements traditional machine learning operations and Software as a Medical Device (SaMD) lifecycle monitoring by integrating technical surveillance with human oversight, organizational learning, and resilience-oriented governance across the AI lifecycle. Importantly, fostering a culture of continuous competency development is essential. Recent frameworks for trustworthy AI deployment, including the TRIAD framework, emphasize workforce education, human oversight, governance, and adaptive implementation as critical enablers of safe and effective clinical AI adoption (Navigating the Trustworthy AI Landscape: Insights from the Open Voice Trustmark Webinar, 2024; Li et al., 2026). AI governance and responsible deployment frameworks increasingly highlight operational literacy as a core pillar of safe AI adoption (Navigating the Trustworthy AI Landscape: Insights from the Open Voice Trustmark Webinar, 2024; Li et al., 2026). Education must extend beyond tool operation to include training on when to question, override, or contextualize AI recommendations—ensuring clinicians maintain cognitive authority and professional accountability in human-AI collaborative environments (Navigating the Trustworthy AI Landscape: Insights from the Open Voice Trustmark Webinar, 2024). These actions move healthcare organizations beyond static compliance toward adaptive, resilience-oriented assurance ecosystems, where governance, technology, and human expertise co-evolve with continuously learning clinical AI systems.

Despite its potential advantages, STRAICS is not without limitations. Continuous assurance frameworks may increase implementation costs, technical complexity, and workforce requirements, potentially creating barriers for smaller healthcare organizations and resource-limited settings. The substantial upfront investment required for monitoring infrastructure, data engineering pipelines, and specialized personnel may be prohibitive for rural hospitals, safety-net institutions, and international healthcare systems with limited informatics capacity. This financial barrier raises concerns that STRAICS adoption could inadvertently exacerbate existing health disparities, as well-resourced institutions gain sophisticated AI governance capabilities while underserved settings struggle to maintain basic model monitoring. Extensive monitoring, documentation, and governance activities may also contribute to audit fatigue and increase administrative burden. Furthermore, overly prescriptive assurance requirements could inadvertently slow innovation by increasing compliance obligations for developers and healthcare institutions. For these reasons, resilience-oriented assurance should be implemented using risk-based and scalable approaches that align monitoring intensity with the clinical impact and risk profile of specific AI applications. Risk stratification should consider the clinical severity of potential AI failures, the vulnerability of target patient populations, and institutional capacity to detect and respond to performance degradation. Future research is needed to evaluate the cost-effectiveness, feasibility, and unintended consequences of continuous assurance models across diverse healthcare environments, with particular emphasis on developing validated, low-cost monitoring approaches suitable for resource-constrained settings and assessing whether differential implementation capacity creates a two-tiered AI safety ecosystem.

## 7 Conclusion

The audit trail for AI-Driven Clinical Decision Support Systems (AI-CDSS), while actively being forged, remains uncharted in its most critical dimensions, particularly those related to system adaptation, emergent behavior, and long-term resilience. The trajectory of the field, however, is becoming increasingly clear. Healthcare has progressed from viewing AI-CDSS as discrete technological artifacts validated at a single point in time toward recognizing them as dynamic components embedded within complex, adaptive socio-technical systems that require continuous, lifecycle-based assurance (Sendak et al., 2020; Hollnagel, 2014; World Health Organization, 2021). The next evolutionary phase must extend beyond even sophisticated continuous monitoring to embrace the proactive cultivation of socio-technical resilience and continuous risk governance. Rather than treating risk assessment as a one-time regulatory exercise performed before deployment, future assurance frameworks should continuously identify, evaluate, and mitigate evolving technical, clinical, organizational, and regulatory risks throughout the AI lifecycle. This requires designing and auditing systems not merely to avoid failure under expected operating conditions, but to demonstrate the intrinsic capacity to adapt safely, learn effectively from real-world deployment, and maintain clinical safety and effectiveness when confronted with unexpected data, workflow variation, or environmental disruption (Hollnagel, 2014; Li et al., 2026; National Academy of Medicine and the Learning Health System Series, 2023).

This transition necessitates a fundamental reconceptualization of the audit function. Resilience engineering emphasizes anticipation, monitoring, response, and learning as core system capabilities rather than focusing solely on error prevention (Hollnagel, 2014; National Academy of Medicine and the Learning Health System Series, 2023). Within healthcare AI, this shift requires the development of novel audit methodologies that integrate technical performance evaluation with social, behavioral, and organizational science insights. It also requires the creation of new measurement frameworks capable of capturing attributes such as graceful degradation, adaptive recovery, and organizational learning velocity following near-misses or adverse events (Wiens et al., 2019; Hollnagel, 2014). Equally important is the need for governance and organizational structures that authentically integrate clinical expertise, data science, ethics, health equity perspectives, and patient representation into AI oversight processes. Contemporary trustworthy AI frameworks emphasize transparency, accountability, fairness, and human oversight as foundational principles, reinforcing the concept that AI safety is an emergent property of the entire healthcare ecosystem rather than of algorithms alone (U.S. Food and Drug Administration, 2021; McCradden et al., 2020; European Commission High-Level Expert Group on AI, 2019).

Most fundamentally, this evolution requires reframing the central question of healthcare AI audit. The objective can no longer be narrowly defined as, “Is the AI system performing correctly relative to its original specification?” Instead, the modern audit mandate must address a broader, system-level question: “Is the healthcare system—now integrally incorporating AI—operating safely, effectively, equitably, and adaptively for all populations it serves?” (World Health Organization, 2021; McCradden et al., 2020) This shift aligns with emerging regulatory and ethical consensus that AI assurance must incorporate real-world performance monitoring, bias surveillance, human-AI interaction quality, and long-term outcome impact across diverse patient populations (U.S. Food and Drug Administration, 2021; McCradden et al., 2020; European Commission High-Level Expert Group on AI, 2019). As artificial intelligence becomes increasingly embedded in the fabric of clinical care, assurance methodologies must evolve with commensurate sophistication, foresight, and human-centered design. By embracing a resilience-oriented paradigm today, healthcare organizations can develop audit practices and, by extension, healthcare delivery systems that are not only technologically advanced but also robust, trustworthy, equitable, and capable of thriving amid the inevitable complexity and uncertainty of future clinical environments (Sendak et al., 2020; Hollnagel, 2014; World Health Organization, 2021; McCradden et al., 2020).

Future research should therefore focus on validating the STRAICS framework across diverse healthcare settings, developing standardized resilience and risk metrics, evaluating cost-effective implementation strategies for resource-limited organizations, and establishing internationally harmonized assurance methodologies for adaptive AI systems. Such efforts will be essential to translate resilience-oriented assurance from a conceptual framework into evidence-based, scalable, and routinely adopted clinical practice.
